# Chagasic meningoencephalitis after heart transplant

**DOI:** 10.1016/j.idcr.2021.e01353

**Published:** 2021-11-27

**Authors:** Jenny Paola Garzón Hernández, Alexander Pabón Moreno, Rafael Esteban González Vesga, Luis Guillermo Uribe Rodríguez, Federico Arturo Silva Sieger

**Affiliations:** a Grupo de Ciencias Neurovasculares, Fundación Cardiovascular de Colombia; Instituto Neurológico, Hospital Internacional de Colombia, Piedecuesta, Colombia; b Universidad Autónoma de Bucaramanga, Colombia; c Unidad de Infectología, Fundación Cardiovascular de Colombia, Hospital Internacional de Colombia, Piedecuesta, Colombia

A 46-year-old male with an orthotopic heart transplant (HTx) and history of chronic Chagas cardiomyopathy presented to our institute with a 7-day history of slurred speech, hypoesthesia on the right side of the body, and unsteady gait. At presentation, he exhibited disorientation, with a clouded mental status, aphasia, left ophthalmoplegia (III-IV cranial nerves), central facial palsy, and right-sided hemiparesis. After admission on day 2, a brain MRI showed an expansive lesion in the frontotemporal cortex with swelling (Fig. 1 A-D). Cerebrospinal fluid (CSF) analysis showed live *Trypanosoma cruzi* trypomastigotes (video). Chagas disease reactivation was finally diagnosed with the visualization of trypomastigotes using both the Strout method in peripheral blood and direct microscopy of the CSF sample. He was administered 200 mg of benzimidazole every 12 h and improved at 3-months after discharge.

**Fig. 1:**
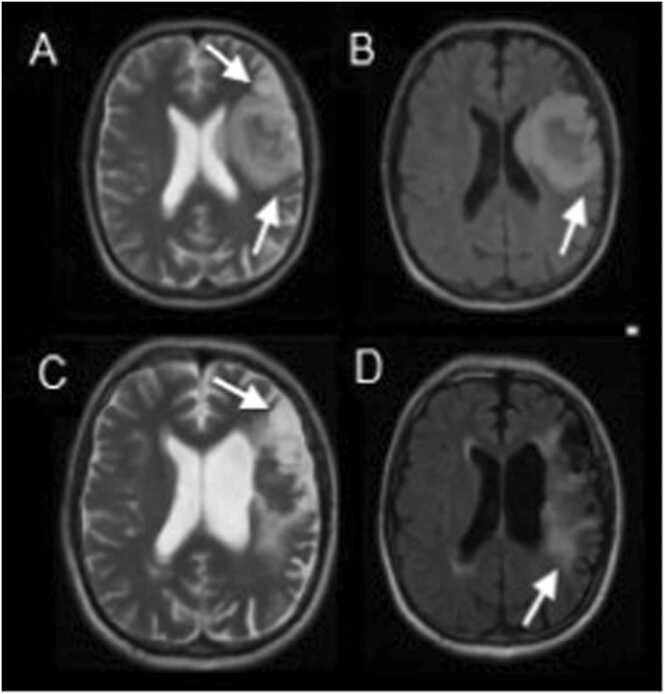
**Axial sections of brain MRI.** (A, C) Brain MRI at 8-months after heart transplant reveals increased signal intensity in the cerebral cortices with swelling on diffusion-weighted images and (B, D) T2-weighted images (arrows).

Supplementary material related to this article can be found online at doi:10.1016/j.idcr.2021.e01353.

The following is the Supplementary material related to this article Video 1..

Video 1 Trypanosoma cruzi trypomastigotes in fresh cerebrospinal fluid.

Chagas reactivation after HTx related to aggressive immunosuppression has been reported. Prevention, early polymerase chain reaction, and treatment approaches in patients with heart transplants should be main recommendations and might reduce mortality.

## Consent

Written informed consent was obtained from the patient for publication of this case report and accompanying images. A copy of the written consent is available for review by the Editor-in-Chief of this journal on request.

## Contributors

JG and EG reviewed the literature and drafted the manuscript. AP, LU and FS did critical review. All authors edited the manuscript and approved the final draft. Written informed consent for publication was obtained from the brother of the patient. Our institute gave IRB approval for submission of the image and video of the patient.

## CRediT authorship contribution statement

Dr. Jenny Garzon: review of the literature, drafting of the manuscript and final approval. Dr Alexander Pabon: critical review and final approval. Dr. Esteban Gonzalez: review of the literature, drafting of the manuscript and final approval. Dr Luis Uribe critical review and final approval. Dr Federico Silva critical review and final approval.

## Declaration of interests

We declare no competing interests.

